# Inflammatory Markers Associated with Antidepressant-Related Side Effects in Patients with Major Depressive Disorder: A Cross-Sectional Study

**DOI:** 10.3390/jcm15187160

**Published:** 2026-09-15

**Authors:** Ezgi Çankaya İnan, Mahmut Bulut, Betül Uyar

**Affiliations:** 1 Department of Psychiatry, Defne State Hospital, 31000 Hatay, Türkiye; ezgcnkya@gmail.com; 2 Department of Psychiatry, Faculty of Medicine, Dicle University, 21280 Diyarbakır, Türkiye; drmahmutbulut@yahoo.com

**Keywords:** antidepressant, side effect, inflammation, depression

## Abstract

**Background**: Major depressive disorder (MDD) has frequently been associated with inflammation; however, the relationship between antidepressant-related side effects and inflammatory markers remains unclear. This study investigated the associations of antidepressant side effects with the neutrophil-to-lymphocyte ratio (NLR), monocyte-to-HDL ratio (MHR), monocyte-to-lymphocyte ratio (MLR), platelet-to-lymphocyte ratio (PLR), white blood cell (WBC) count, and C-reactive protein (CRP). **Methods**: The study included 60 patients aged 18–65 years who met DSM-5 criteria for MDD, had used antidepressants for at least one month, and developed side effects, together with 60 sociodemographically matched healthy controls. Complete blood count (CBC), CRP, and HDL levels were measured from venous blood samples, and NLR, MHR, MLR, and PLR were calculated. Patients completed the Udvalg Kliniske Undersøgelser (UKU) Side Effect Rating Scale, Clinical Global Impression Scale, Montgomery–Åsberg Depression Rating Scale (MADRS), and Hamilton Anxiety Scale (HAM-A). Only side effects considered medication-related according to the UKU causality assessment were included. Analyses were performed using SPSS 24.0. **Results**: Concentration problems correlated negatively with neutrophil levels and NLR. Sedation/drowsiness correlated positively with MHR and MLR, and increased dreaming correlated positively with platelet levels. Weight gain correlated positively with neutrophil, lymphocyte, and WBC counts and negatively with PLR. Erectile dysfunction correlated negatively with PLR, orgasmic dysfunction with monocyte levels, vaginal dryness with neutrophil levels, and physical dependence with NLR. **Conclusions**: Antidepressant-related side effects showed several uncorrected associations with peripheral inflammatory markers, none of which survived correction for multiple comparisons (Benjamini–Hochberg false discovery rate). As the first exploratory investigation of this relationship, these preliminary, hypothesis-generating findings may inform understanding of side-effect mechanisms and support future, adequately powered longitudinal studies aimed at enhancing treatment adherence and reducing relapse and chronicity.

## 1. Introduction

Major Depressive Disorder (MDD) is characterized by depressed mood, anhedonia, and related symptoms—including changes in appetite and sleep, feelings of guilt or worthlessness, and thoughts of death—persisting for at least two weeks, with a marked tendency toward relapse and chronicity [1]. MDD is approximately twice as common in women as in men, has a lifetime prevalence of 5–17%, and is one of the most common psychiatric disorders [2], ranking as the third leading cause of disability worldwide, with prevalence rising over the past 10–15 years [3]. The World Health Organization has projected that depression may become the leading contributor to the global disease burden by 2030, underscoring the need for a better understanding of its underlying mechanisms and treatment-related outcomes [4].

Although the neurobiology of MDD has been extensively studied, the mechanisms underlying the disorder remain incompletely understood. Activation of inflammatory pathways is thought to play a key role in its onset [5]; stress-related elevations in proinflammatory cytokines can induce depressive symptoms through changes in neuroendocrine signaling and neurotransmitter metabolism, and antidepressants appear to normalize cytokine levels in parallel with symptomatic improvement [6]. Depression has also been linked to increased leukocyte and neutrophil counts, with the neutrophil-to-lymphocyte ratio (NLR) established as a reliable, low-cost marker of systemic inflammation, while the platelet-to-lymphocyte ratio (PLR), monocyte-to-lymphocyte ratio (MLR), and monocyte-to-HDL ratio (MHR) are similarly used to assess peripheral inflammatory status in a range of medical and psychiatric conditions [7,8].

Psychotherapy, neuromodulation, and pharmacotherapy are all effective treatment modalities in MDD [9,10], and current guidelines recommend antidepressants as first-line treatment [11,12]. Antidepressant classes in clinical use include tricyclics, selective serotonin reuptake inhibitors, serotonin-norepinephrine reuptake inhibitors, norepinephrine reuptake inhibitors, and monoamine oxidase inhibitors, among others. However, antidepressant use is frequently accompanied by side effects—including gastrointestinal symptoms, sexual dysfunction, weight change, tremor, apathy, headache, and insomnia—that are well documented in the literature. These side effects reduce treatment adherence, which can in turn lead to treatment failure, relapse, chronicity of depression, additional complications, increased healthcare costs, lost productivity, and broader impairment in daily functioning [13,14].

Despite this well-established clinical burden, no previous study has examined whether interindividual variability in antidepressant side effects is related to systemic inflammatory activity, even though both depression itself and several of its common side effects (e.g., sedation, weight change, sexual dysfunction) plausibly intersect with inflammatory pathways. This study therefore investigated the relationship between antidepressant-related side effects and peripheral inflammatory markers—NLR, MHR, MLR, PLR, WBC count, and CRP—in patients with MDD who developed side effects after at least one month of antidepressant treatment, with the aim of clarifying whether inflammatory activity contributes to the individual variability observed in treatment tolerability.

## 2. Materials and Methods

### 2.1. Sample Selection

The study included 60 patients diagnosed with MDD according to DSM-5 criteria who had been using antidepressant medication for at least one month, along with 60 healthy volunteers. All participants provided written informed consent before enrollment. The study received approval from the Ethics Committee for Non-Interventional Clinical Research of Dicle University Faculty of Medicine, with protocol number 65, dated 28 February 2023.

### 2.2. Inclusion Criteria

Participants were required to meet the following criteria: a diagnosis of MDD according to DSM-5 criteria, ongoing antidepressant treatment for at least one month, the presence of side effects, an age range of 18–65 years, voluntary participation, and the ability to provide informed consent.

### 2.3. Exclusion Criteria

The study excluded participants with any of the following conditions: neurological disorders, inflammatory or autoimmune diseases, chronic illnesses such as hypertension or diabetes mellitus, a history of head or spinal trauma, intellectual disability, pregnancy, active infections, substance or alcohol dependence, or the use of medications other than psychiatric drugs.

### 2.4. Assessment Tools

All participants completed a sociodemographic data form. The research team assessed the patient group using the Udvalg Kliniske Undersøgelser (UKU) Side Effect Rating Scale, the Clinical Global Impression Scale (CGI), the Montgomery–Åsberg Depression Rating Scale (MADRS), and the Hamilton Anxiety Scale (HAM-A). We excluded patients who did not develop side effects according to the UKU scale from the statistical analysis. The causality parameter of the UKU scale evaluated the relationship between side effects and medication, and we included only medication-related side effects in the analysis. Additionally, the study collected venous blood samples from both the patient and control groups to analyze inflammatory markers.

#### 2.4.1. Sociodemographic Data Form

This form, designed to evaluate participants’ sociodemographic characteristics, includes information on age, gender, educational level, marital status, employment status, personal and family medical history, and alcohol or substance use.

#### 2.4.2. UKU Side Effect Rating Scale

Developed by Lingjaerde et al. in 1987, this scale evaluates the side effects associated with the use of psychotropic drugs by establishing a causal relationship [15,16]. It assesses the type, severity, and affected areas of side effects caused by medications [16]. Each UKU item is rated by the clinician on a four-point ordinal severity scale (0 = not present, 1 = mild, 2 = moderate, 3 = severe), and a separate causality rating classifies whether the item is probably, possibly, or not related to the current medication. For the primary analysis, only items rated as medication-related on the causality parameter were retained, and for these retained items the ordinal severity score (0–3) for each patient who endorsed that side effect was entered as the correlate in the Spearman analyses; patients who did not endorse a given side effect did not contribute a value for that item. We treated the 0–3 ordinal severity score as suitable for Spearman rank-order correlation, which does not require interval-level measurement, consistent with prior UKU-based studies.

#### 2.4.3. Clinical Global Impression Scale (CGI)

This three-dimensional scale, developed by Guy et al., evaluates the severity of the illness, the patient’s response to treatment, and adherence to therapy [17]. This study included the sections “Severity of Illness” and “Efficacy Index” in the analysis.

#### 2.4.4. The Montgomery–Åsberg Depression Rating Scale (MADRS)

This scale measures the level and severity of core depressive symptoms in patients. It consists of a total of 10 items, with each item scored on a scale from 0 to 6. The Turkish validity and reliability study was conducted by Özer et al. (2001) [18].

#### 2.4.5. Hamilton Anxiety Scale (HAM-A)

This scale, developed by Hamilton et al. in 1956, consists of 14 items and aims to assess anxiety’s psychological and somatic symptoms. The Turkish validity and reliability study was conducted by Yazıcı et al. (1998) [19].

#### 2.4.6. Blood Sampling and Laboratory Measurements

CRP, HDL, and CBC were measured using venous blood samples after a 12 h fasting period. The central laboratory of Dicle University Faculty of Medicine Hospital conducted the analysis.

### 2.5. Institutional Review Board Statement

The study was conducted in accordance with the Declaration of Helsinki and approved by the Ethics Committee for Non-Interventional Clinical Research of Dicle University Faculty of Medicine (protocol code 65; 28 February 2023).

### 2.6. Statistical Analysis

Statistical analyses were performed using SPSS for Windows, version 24.0 (SPSS Inc., Chicago, IL, USA). Descriptive statistics were expressed as frequency, percentage, mean, standard deviation, and median. The chi-square test was used to analyze categorical variables. The Kolmogorov–Smirnov test was used to assess the normality of the distribution of numerical variables. The independent samples *t*-test was used for normally distributed variables, and the Mann–Whitney U test was used for non-normally distributed variables. The Spearman correlation test was used to examine relationships between numerical variables. The Benjamini–Hochberg procedure was applied to control the false discovery rate across the family of correlation tests. A *p*-value of <0.05 was considered statistically significant.

## 3. Results

### 3.1. Comparison of Sociodemographic Characteristics

Table 1 shows the sociodemographic characteristics of the patient and control groups. Since we matched the groups for sociodemographic characteristics, we found no significant differences in age, BMI, gender, education level, marital status, employment status, or smoking habits (*p* > 0.05) but a higher family history of psychiatric disorders in the patient group (*p* = 0.011).

### 3.2. Correlation Analysis Results Between Side Effects and Biochemical Parameters

Table 2 presents the correlation analysis results between the inflammatory parameters (WBC, Neutrophil, Lymphocyte, Monocyte, Platelet counts, HDL, CRP, PLR, MHR, MLR, NLR) and the sub-parameters of the UKU Side Effect Rating Scale in patients who developed drug-related side effects due to antidepressant use.

Concentration difficulties showed a significant negative correlation with both neutrophil counts and NLR (*p* = 0.013). Sleepiness/sedation demonstrated significant positive correlations with MHR (*p* = 0.039) and MLR (*p* = 0.034). An increase in dreaming showed a significant positive correlation with platelet counts (*p* = 0.014). Weight gain positively correlated with neutrophil, lymphocyte, and WBC counts, while it negatively correlated with PLR. (*p* = 0.016, *p* = 0.019, *p* < 0.001, *p* = 0.012). Erectile dysfunction exhibited a significant negative correlation with PLR (*p* = 0.028). Orgasmic dysfunction showed a significant negative correlation with monocyte counts (*p* = 0.048). Vaginal dryness demonstrated a significant negative correlation with neutrophil counts (*p* = 0.038). Finally, physical dependence had a significant negative correlation with NLR (*p* = 0.023).

After applying the Benjamini–Hochberg procedure to control the false discovery rate across the 176 correlations examined in Table 2, none of the nominally significant associations described above remained statistically significant (all FDR-adjusted q ≥ 0.088). This indicates that, while several uncorrected correlations reached the conventional *p* < 0.05 threshold, none are robust to correction for multiple comparisons, and the associations reported here should be interpreted as preliminary, hypothesis-generating observations rather than confirmed findings.

Correlation analysis did not reveal any significant relationships between the side effects of weakness/fatigue/easy fatigability, forgetfulness, prolonged sleep duration, emotional indifference, tremor, reduced salivation, decreased sexual desire, and the impact of drug side effects on patients’ daily performance, and inflammatory markers.

## 4. Discussion

Before interpreting the individual associations below, it is important to state explicitly that this is a cross-sectional study and, as such, cannot establish causality or temporal direction between antidepressant-related side effects and peripheral inflammatory markers. The correlations we observed indicate that a given side effect and a given inflammatory marker were measured concurrently and were statistically associated at the nominal *p* < 0.05 level; they do not indicate that the inflammatory change caused the side effect, that the side effect caused the inflammatory change, or that either precedes the other in time. Furthermore, as reported in the Results, none of these associations remained significant after correction for multiple comparisons. Accordingly, the interpretations offered in this section are framed as hypotheses warranting confirmation in future longitudinal studies rather than as mechanistic conclusions.

Poor treatment adherence due to side effects is one of the main barriers to achieving remission in MDD [20]. In our sample, the most frequently reported side effects included concentration difficulties, fatigue/easy exhaustion, drowsiness/sedation, forgetfulness, prolonged sleep duration, increased dreaming, emotional blunting, reduced libido, erectile dysfunction, weight gain, and vaginal dryness. Identifying objective biological correlates of these side effects could help clinicians anticipate tolerability problems and select antidepressants more rationally, which is the broader motivation for the associations examined below.

Concentration difficulties correlated negatively with neutrophil count and NLR. This relationship has not previously been studied in MDD, but elevated NLR has been linked to cognitive impairment [21] and postoperative cognitive dysfunction [22], and neutrophils are implicated in Alzheimer’s disease pathogenesis [23]. In contrast, ADHD studies have found no significant differences in inflammatory markers, including NLR, PLR, MLR, and CRP, versus healthy controls [24,25], with such null findings generally attributed to the disorder’s neurodevelopmental pathogenesis. Because our study evaluated concentration difficulties strictly as a drug-induced side effect, antidepressant-induced concentration difficulties may involve inflammatory mechanisms distinct from those seen in ADHD or classical cognitive impairment.

Sedation and drowsiness correlated positively with MHR and MLR. Although this specific association has not been examined in MDD, higher sleep quality has been linked to lower MHR in healthy women [26], and individuals reporting daytime sleepiness have shown higher CRP levels in prior work, albeit without controlling for pre-existing illness or hypnotic medication use [27]. Because our study explicitly excluded participants with comorbid medical conditions and those using non-antidepressant medications, it is plausible to hypothesize that antidepressant-induced sedation itself, rather than confounding illness, contributes to the increased MHR and MLR observed in our sample; however, as this is a cross-sectional, uncorrected association, this interpretation remains speculative and requires confirmation in a longitudinal design that can establish temporal precedence.

Increased dreaming correlated positively with platelet levels. The relationship between increased dreaming and inflammatory markers has not been extensively explored in MDD, but comparable links between REM-sleep-related phenomena and inflammation have been reported in Parkinson’s disease, where REM sleep behavior disorder was associated with CRP and MLR [28] and with monocyte levels [29]. These prior findings are generally interpreted within the context of neurodegenerative disease pathogenesis. We propose instead that antidepressant-induced increased dreaming may relate more directly to platelet activation as a pharmacological effect.

Weight gain correlated positively with neutrophil, lymphocyte, and WBC counts, and negatively with PLR. This pattern is broadly consistent with prior reports linking obesity to leukocytosis [30,31] and to changes in platelet indices [32]. We note that obesity was not itself a formal exclusion criterion in this study; BMI did not differ significantly between the patient and control groups (Table 1), but individual BMI was not entered as a covariate in the correlation analyses, so a partial contribution of adiposity-related inflammation to this association cannot be excluded. With this caveat, we consider it plausible, though unconfirmed, that antidepressant-induced weight gain itself contributes to the observed association rather than pre-existing obesity-related inflammation alone. Unlike much of the prior literature on obesity, we found no significant association between weight gain and either CRP or platelet count, suggesting that these two markers may not be central to the specific mechanism of antidepressant-induced weight gain.

Erectile dysfunction (ED) correlated negatively with PLR. Numerous prior studies link ED to elevated NLR and PLR [33,34,35], and to CRP and other inflammatory markers [36], generally attributing these associations to the vascular and endothelial pathophysiology underlying ED. In our cohort, however, ED was evaluated strictly as an antidepressant-induced side effect rather than as a primary vascular condition, and was instead associated with a reduction in PLR. This discordant direction of association suggests that drug-induced ED may involve a mechanism distinct from vasculogenic ED and warrants dedicated mechanistic study.

Orgasmic dysfunction correlated negatively with monocyte levels, an association that, to our knowledge, has not been previously reported. A related study of premature ejaculation found a positive association with HDL cholesterol and with visceral adiposity, again framed within a metabolic-disease context rather than a medication-effect one [37]. Our finding suggests that antidepressant-induced orgasmic dysfunction may instead be associated with a reduction in circulating monocytes.

Vaginal dryness correlated negatively with neutrophil count. The relationship between vaginal dryness and inflammation has not previously been examined in MDD. One study in the general population linked reduced vaginal arousal to higher CRP, interpreting high inflammation as potentially detrimental to sexual function, but that study excluded users of psychotropic medication [38], unlike our sample. Based on our findings, vaginal dryness as a side effect of antidepressant use may instead involve a decrease in neutrophil activity rather than a CRP-driven inflammatory process.

Physical dependence (withdrawal symptoms) correlated negatively with NLR. Antidepressant discontinuation syndrome is thought to reflect neuroadaptation to chronic drug exposure and disruption of a newly established homeostatic balance, rather than classical addiction, with risk increasing with higher doses, longer treatment duration, and specific antidepressant agents [39]. Although we did not assess treatment duration or dosage in this study, withdrawal symptoms may have varied proportionally with these factors. No prior study has examined the inflammatory correlates of antidepressant withdrawal specifically, though NLR and PLR elevations have been reported in heroin [40] and cocaine [41] use disorders. Because the antidepressants used by our patients do not act on opioid receptor pathways, discordant findings relative to the substance-use-disorder literature are not unexpected.

It is worth noting that several of the key associations reported above—concentration problems with neutrophils and NLR, erectile dysfunction with PLR, orgasmic dysfunction with monocytes, vaginal dryness with neutrophils, and physical dependence with NLR—were negative correlations, meaning that lower inflammatory marker values were associated with greater side-effect burden. This pattern runs counter to the more intuitive expectation that higher inflammation would track with greater symptom severity, and we did not initially address it in detail. Several non-mutually exclusive explanations merit consideration. First, compensatory or counter-regulatory immune shifts are recognized in chronic psychotropic exposure, whereby an initial inflammatory response may be followed by a rebound suppression of specific leukocyte subpopulations; a lower neutrophil or monocyte count could therefore reflect a later phase of this dynamic rather than an absence of inflammatory involvement. Second, the timing of venous sampling relative to symptom onset and medication dosing was not standardized in this cross-sectional design, and inflammatory markers such as NLR and PLR fluctuate over the course of a day and across the treatment timeline; a single measurement may capture a trough rather than a peak. Third, the UKU side-effect categories are heterogeneous phenomenologically (for example, concentration problems and vaginal dryness likely reflect different underlying pharmacological pathways), so a uniform direction of association across all side effects may not be biologically expected. We present these as candidate explanations for future longitudinal work rather than as established mechanisms, particularly given that none of these associations survived correction for multiple comparisons.

No significant correlations were found between inflammatory markers and fatigue/easy exhaustion, forgetfulness, prolonged sleep duration, emotional blunting, tremor, reduced salivation (dry mouth), or decreased libido. Prior literature on these symptoms is mixed and largely derived from disease-related rather than drug-induced contexts: CRP has been linked to depressive fatigue [42] and to sleep duration in a U-shaped pattern [43], findings on apathy and CRP are inconsistent [44], and Sjögren’s-syndrome-related dry mouth has been linked to elevated NLR and PLR in a disease-specific context [45]. Because these earlier associations largely reflect disease pathogenesis in older or medically comorbid populations rather than medication effects in a younger, otherwise healthy cohort, the absence of correlation here may indicate a distinct, non-inflammatory mechanism, or insufficient power to detect a smaller effect.

### 4.1. Strengths of the Study

This study has notable strengths. We excluded major confounders of inflammatory status—comorbid medical illness, substance or alcohol use, non-psychiatric medication, pregnancy, active infection, head or spinal trauma, and extreme age—and assessed causality using a validated, standardized scale, which strengthens the objectivity of the side-effect assessment relative to studies that do not formally establish drug causality. To our knowledge, this is the first study to examine the relationship between antidepressant-related side effects and peripheral inflammatory markers, and as such it may serve as a foundation for future mechanistic and confirmatory research in this area.

### 4.2. Limitations of the Study

This study has several limitations. The single-center design and modest sample size restricted evaluation of less common side effects, and multi-center studies with larger samples are needed. The cross-sectional design did not allow serial measurement of inflammatory parameters before and after antidepressant initiation. Antidepressant class, dose, and treatment duration beyond the one-month minimum were not recorded, precluding class-stratified analyses. Multivariable models adjusting for BMI, smoking status, depression and anxiety severity, and family psychiatric history were not constructed, and per-item sample sizes for the UKU correlations, which were smallest for sex-specific side effects, were not reported. Descriptive statistics for CRP and HDL were not presented separately. Controls were not screened with a structured interview for subthreshold psychiatric symptoms, and the control group served as a sociodemographic reference rather than for a direct case–control comparison of inflammatory markers. Future prospective, multi-center studies with covariate-adjusted analyses are needed to address these limitations.

## 5. Conclusions

The findings of our study can be summarized as follows:

(1) Concentration problems were significantly negatively correlated with neutrophil count and NLR.
(2) Sedation and drowsiness significantly correlated with MHR and MLR.
(3) Increased dreaming was significantly positively correlated with platelet levels.
(4) Weight gain was significantly positively correlated with neutrophil count, lymphocyte count, and WBC levels while showing a significant negative correlation with PLR.
(5) Erectile dysfunction demonstrated a significant negative correlation with PLR.
(6) Orgasm disorder was significantly negatively correlated with monocyte levels, while vaginal dryness showed a significantly negative correlation with neutrophil count.
(7) Physical dependence demonstrated a significant negative correlation with NLR.
(8) No significant association was observed between inflammatory parameters and the MADRS or CGI scores of depression severity, indicating that the inflammatory parameters in MDD patients who experience side effects are not related to the severity of depression.

These findings suggest that, among antidepressant-treated patients who had already developed side effects, the severity of some of those side effects correlated with selected peripheral inflammatory markers; because all patients in this study had side effects by design, these data cannot establish whether inflammatory markers predict which patients will go on to develop side effects, and we no longer frame the findings in predictive terms. One of the most significant issues in the medical field is preventing drug side effects, which may be achievable through a clearer understanding of their mechanisms. The relationships we observed between antidepressant side effects and inflammatory markers may serve as a foundation for future studies and contribute to clarifying the mechanisms of these side effects. Concretely, we suggest that future prospective studies measure NLR, MHR, MLR, PLR, WBC, and CRP before antidepressant initiation and again after side effects emerge, in patients with and without side effects, to test directly whether baseline or early-treatment inflammatory profiles are associated with subsequent tolerability; if replicated, such markers could eventually inform a low-cost, blood-based component of a clinical decision aid for antidepressant selection, to be used alongside, not in place of, established clinical judgment. Our study is the first, to our knowledge, to examine this relationship, and its findings should be regarded as hypothesis-generating.

## Figures and Tables

**Table 1:** Comparison of sociodemographic characteristics between the patient and healthy control groups.

	Patient Group (*n* = 60) M ± SD	Control Group (*n* = 60) M ± SD	*p* ^+^
Age	33.5 ± 10.3	34.1 ± 9.0	0.757
BMI	25.2 ± 4.6	24.8 ± 3.0	0.546
	*n* (%)	*n* (%)	*p^++^*
Gender			0.843
Female	42 (70.0)	41 (68.3)	
Male	18 (30.0)	19 (31.7)	
Educational Level			(*p* > 0.05)
Illiterate	2 (3.3)	1 (1.7)	
Literate	5 (8.3)	3 (5.0)	
Primary	9 (15)	8 (13.3)	
Secondary	22 (36.7)	22 (36.7)	
University	22 (36.7)	26 (43.3)	
Marital Status			0.853
Single	26 (43.3)	25 (41.7)	
Married	34 (56.7)	35 (58.3)	
Employment Status			(*p* > 0.05)
Unemployed	27 (45.0)	20 (33.3)	
Employed	33 (55.0)	40 (66.7)	
Smoking Status			0.707
No	38 (63.3)	36 (60.0)	
Yes	22 (36.7)	24 (40.0)	
Family History			0.011 *
Absent	34 (56.7)	47 (78.3)	
Present	26 (43.3)	13 (21.7)	

***p*+:** Mann–Whitney U test statistical significance value, ***p*++:** Chi-square test statistical significance value, **M:** Mean, **SD:** Standard Deviation, ***n:*** count, **BMI:** Body Mass Index, *: Statistically significant difference at *p* < 0.05.

**Table 2:** Correlation analysis results between side effects and biochemical parameters.

		Neutrophil	Lymphocyte	Platelet	HDL	Monocyte	WBC	CRP	PLR	MHR	MLR	NLR
**UKU**	**r**	−0.510 *	−0.075	−0.293	−0.023	−0.248	−0.309	−0.23	−0.14	−0.078	−0.182	−0.512 *
**Psychol.1**	***p***	0.013	0.734	0.174	0.918	0.253	0.152	0.29	0.523	0.722	0.405	0.013
**UKU**	**r**	−0.188	0.113	0.17	0.16	0.129	0.021	−0.27	0	0.075	0.025	−0.248
**Psychol.2**	***p***	0.357	0.583	0.406	0.434	0.529	0.92	0.183	1	0.715	0.903	0.222
**UKU**	**r**	−0.231	−0.257	−0.163	−0.143	0.183	−0.158	0.244	0.209	0.303 *	0.310 *	−0.051
**Psychol.3**	***p***	0.117	0.081	0.274	0.339	0.218	0.29	0.098	0.159	0.039	0.034	0.734
**UKU**	**r**	0.222	0.258	0.166	−0.111	−0.185	0.295	0.074	−0.185	−0.111	−0.332	0.074
**Psychol.4**	***p***	0.334	0.258	0.471	0.632	0.423	0.194	0.75	0.423	0.632	0.141	0.75
**UKU**	**r**	−0.01	0.003	0.071	0.193	−0.163	−0.001	−0.04	0.051	−0.218	−0.096	−0.036
**Psychol.7**	***p***	0.951	0.983	0.647	0.21	0.291	0.997	0.817	0.741	0.155	0.537	0.816
**UKU**	**r**	0.006	−0.089	0.368 *	0.177	−0.19	−0.043	0.031	0.246	−0.233	−0.105	0.089
**Psychol.9**	***p***	0.972	0.567	0.014	0.251	0.216	0.78	0.844	0.108	0.128	0.497	0.564
**UKU**	**r**	0.043	−0.02	0.255	0.13	−0.096	0.054	−0.16	0.109	−0.068	0.051	0.032
**Psychol.10**	***p***	0.782	0.897	0.095	0.399	0.534	0.728	0.305	0.481	0.659	0.743	0.839
**UKU**	**r**	0.497	0.321	0.284	−0.285	−0.426	0.569	0.142	−0.142	−0.284	−0.497	0.071
**Neurol.5**	***p***	0.143	0.366	0.426	0.425	0.219	0.086	0.695	0.695	0.426	0.143	0.845
**UKU**	**r**	0.107	−0.069	−0.156	−0.041	0.014	−0.218	−0.08	−0.018	−0.012	0.179	0.121
**Autonomic3**	***p***	0.693	0.801	0.565	0.88	0.958	0.418	0.758	0.946	0.966	0.507	0.657
**UKU**	**r**	0.404 *	0.394 *	0.077	−0.049	0.223	0.579 **	0.156	−0.421 *	0.184	0.022	0.177
**Other5**	***p***	0.016	0.019	0.662	0.78	0.199	<0.001	0.372	0.012	0.29	0.899	0.31
**UKU**	**r**	−0.091	0.126	−0.008	−0.079	−0.142	−0.041	−0.22	−0.107	−0.045	−0.268	−0.158
**Other12**	***p***	0.604	0.47	0.964	0.652	0.416	0.813	0.211	0.543	0.796	0.119	0.365
**UKU**	**r**	0.195	0.443	−0.018	0.213	0	0.089	0.053	−0.585 *	−0.053	−0.514	−0.053
**Other13**	***p***	0.504	0.113	0.952	0.464	1	0.763	0.857	0.028	0.857	0.06	0.857
**UKU**	**r**	−0.355	−0.168	−0.122	−0.017	−0.427 *	−0.349	0.15	0.229	−0.259	−0.083	−0.096
**Other15**	***p***	0.105	0.455	0.589	0.941	0.048	0.111	0.505	0.305	0.245	0.714	0.671
**UKU**	**r**	−0.493 *	−0.017	−0.287	−0.179	−0.4	−0.438	0.35	0.065	−0.191	−0.273	−0.33
**Other16**	***p***	0.038	0.947	0.248	0.476	0.1	0.069	0.154	0.797	0.447	0.273	0.181
**UKU**	**r**	−0.276	0.17	−0.07	0.039	−0.076	−0.124	−0.14	−0.237	−0.003	−0.146	−0.358 *
**Other18**	***p***	0.085	0.295	0.669	0.809	0.642	0.445	0.38	0.142	0.985	0.37	0.023
**UKU**	**r**	−0.011	0.032	−0.001	−0.069	−0.066	0.016	0.198	0.013	0.029	−0.112	0.041
**Functional Impairment**	***p***	0.933	0.809	0.992	0.598	0.615	0.902	0.13	0.923	0.828	0.396	0.754

**NLR:** Neutrophil to Lymphocyte Ratio, **PLR:** Platelet to Lymphocyte Ratio, **MLR**: Monocyte to Lymphocyte Ratio, **MHR:** Monocyte to HDL Ratio, **WBC:** White Blood Cells, **CRP:** C-reactive Protein, **UKU:** Udvalg Kliniske Undersøgelser Side Effect Rating Scale, ***p*:** Spearman correlation significance value, **r:** Spearman correlation coefficient (0.30 or less indicates weak correlation, 0.30–0.70 indicates moderate correlation, more significant than 0.70 indicates strong correlation). *: * Statistically significant difference at *p* < 0.05. **: * Statistically significant difference at *p* < 0.01. **−:** Negative correlation. **Psychological 1:** Concentration problems, **Psychological 2**: Weakness/fatigue/tiredness, **Psychological 3:** Sedation, **Psychological 4:** Forgetfulness, **Psychological 7:** Increased sleep duration, **Psychological 9:** Increased dreams, **Psychological 10:** Emotional blunting, **Neurological 5:** Tremor, **Autonomic 3:** Reduced salivation, **Other 5:** Weight gain, **Other 12:** Reduced sexual desire, **Other 13**: Erectile dysfunction, **Other 15:** Orgasmic dysfunction, **Other 16:** Vaginal dryness, **Other 18:** Physical dependence (withdrawal symptoms).

## Data Availability

The data supporting the findings of this study are available from the corresponding author upon reasonable request. The data are not publicly available because of participant privacy and ethical restrictions.

## References

[1] American Psychiatric Association (2013). Diagnostic and Statistical Manual of Mental Disorders: DSM-5.

[2] Sadock B.J. (2015). Kaplan & Sadock’s Synopsis of Psychiatry: Behavioral Sciences/Clinical Psychiatry.

[3] James S.L., Abate D., Abate K.H., Abay S.M., Abbafati C., Abbasi N., Abbastabar H., Abd-Allah F., Abdela J., Abdelalim A. (2018). Global, regional, and national incidence, prevalence, and years lived with disability for 354 diseases and injuries for 195 countries and territories, 1990–2017: A systematic analysis for the Global Burden of Disease Study 2017. Lancet.

[4] World Health Organization (2011). Global Burden of Mental Disorders and the Need for a Comprehensive, Coordinated Response from Health and Social Sectors at the Country Level: Report by the Secretariat.

[5] Gardner A., Boles R.G. (2011). Beyond the serotonin hypothesis: Mitochondria, inflammation and neurodegeneration in major depression and affective spectrum disorders. Prog. Neuro-Psychopharmacol. Biol. Psychiatry.

[6] Dowlati Y., Herrmann N., Swardfager W., Liu H., Sham L., Reim E.K., Lanctôt K.L. (2010). A meta-analysis of cytokines in major depression. Biol. Psychiatry.

[7] Demir S., Demir S., Bulut M., İbiloğlu A.O., Güneş M., Kaya M.C., Demirpençe Ö., Sır A. (2015). Neutrophil–lymphocyte ratio in patients with major depressive disorder undergoing no pharmacological therapy. Neuropsychiatr. Dis. Treat..

[8] Marazziti D., Torrigiani S., Carbone M.G., Mucci F., Flamini W., Ivaldi T., Dell’OSso L. (2022). Neutrophil/lymphocyte, platelet/lymphocyte, and monocyte/lymphocyte ratios in mood disorders. Curr. Med. Chem..

[9] Cipriani A., Furukawa T.A., Salanti G., Chaimani A., Atkinson L.Z., Ogawa Y., Leucht S., Ruhe H.G., Turner E.H., Higgins J.P.T. (2018). Comparative efficacy and acceptability of 21 antidepressant drugs for the acute treatment of adults with major depressive disorder: A systematic review and network meta-analysis. Focus.

[10] Gartlehner G., Wagner G., Matyas N., Titscher V., Greimel J., Lux L., Gaynes B.N., Viswanathan M., Patel S., Lohr K.N. (2017). Pharmacological and non-pharmacological treatments for major depressive disorder: Review of systematic reviews. BMJ Open.

[11] Fava M., Rush A.J., Trivedi M.H., Nierenberg A.A., Thase M.E., Sackeim H.A., Quitkin F.M., Wisniewski S., Lavori P.W., Rosenbaum J.F. (2003). Background and rationale for the sequenced treatment alternatives to relieve depression (STAR* D) study. Psychiatr. Clin..

[12] Jobst A., Brakemeier E.-L., Buchheim A., Caspar F., Cuijpers P., Ebmeier K., Falkai P., van der G.R.J., Gaebel W., Herpertz S. (2016). European Psychiatric Association Guidance on psychotherapy in chronic depression across Europe. Eur. Psychiatry.

[13] Cipriani A., Salanti G., Furukawa T.A., Egger M., Leucht S., Ruhe H.G., Turner E.H., Atkinson L.Z., Chaimani A., Higgins J.P.T. (2018). Antidepressants might work for people with major depression: Where do we go from here?. Lancet Psychiatry.

[14] Trivedi M.H., Rush A.J., Wisniewski S.R., Nierenberg A.A., Warden D., Ritz L., Norquist G., Howland R.H., Lebowitz B., McGrath P.J. (2006). Evaluation of outcomes with citalopram for depression using measurement-based care in STAR* D: Implications for clinical practice. Am. J. Psychiatry.

[15] Lindström E., Lewander T., Malm U., Malt U.F., Lublin H., Ahlfors U.G. (2001). Patient-rated versus clinician-rated side effects of drug treatment in schizophrenia. Clinical validation of a self-rating version of the UKU Side Effect Rating Scale (UKU-SERS-Pat). Nord. J. Psychiatry.

[16] Lingjærde O., Ahlfors U.G., Bech P., Dencker S.J., Elgen K. (1987). The UKU side effect rating scale: A new comprehensive rating scale for psychotropic drugs and a cross-sectional study of side effects in neuroleptic-treated patients. Acta Psychiatr. Scand..

[17] Guy W. (1976). ECDEU Assessment Manual for Psychopharmacology.

[18] Özer S.K., Demir B., Tuğal Ö., veKabakçı E. (2001). Montgomery-Asberg Depresyon Değerlendirme Ölçeği: Değerlendiriciler arası güvenilirlik ve geçerlik çalışması. Türk Psikiyatr. Derg..

[19] Yazici M.K., Demir B., Tanriverdi N., Karaagaoglu E., Yolaç P. (1998). Hamilton anksiyete değerlendirme öiçeği, değerlendiriciler arası güvenirlik ve geçerlik çalışması. Türk Psikiyatr. Derg..

[20] SanCar M., DÜzgÜn E., OKuyan B., DEniz S., ÇalIŞKan M., COŞKun K., İzzettin F.V. (2017). Antidepresan Kullanan Majör Depresyon Hastalarında Yan Etkilerin ve Hasta Uyuncunun Değerlendirilmesi. Marmara Pharm. J..

[21] Liu J.-H., Zhang Y.-J., Ma Q.-H., Sun H.-P., Xu Y., Pan C.-W. (2020). Elevated blood neutrophil to lymphocyte ratio in older adults with cognitive impairment. Arch. Gerontol. Geriatr..

[22] Yong R., Meng Y. (2020). Preoperative neutrophil-lymphocyte ratio, an independent risk factor for postoperative cognitive dysfunction in elderly patients with gastric cancer. Geriatr. Gerontol. Int..

[23] Dong Y., Lagarde J., Xicota L., Corne H., Chantran Y., Chaigneau T., Crestani B., Bottlaender M., Potier M., Aucouturier P. (2018). Neutrophil hyperactivation correlates with Alzheimer’s disease progression. Ann. Neurol..

[24] Ceyhun H.A., Gürbüzer N. (2022). New hematological parameters as inflammatory biomarkers: Systemic immune inflammation index, platerethritis, and platelet distribution width in patients with adult attention deficit hyperactivity disorder. Adv. Neurodev. Disord..

[25] Misiak B., Wójta-Kempa M., Samochowiec J., Schiweck C., Aichholzer M., Reif A., Samochowiec A., Stańczykiewicz B. (2022). Peripheral blood inflammatory markers in patients with attention deficit/hyperactivity disorder (ADHD): A systematic review and meta-analysis. Prog. Neuro-Psychopharmacol. Biol. Psychiatry.

[26] Doğan H., Çaltekin M.D. (2021). Sağlikli Kadinlarda Fiziksel Aktivite Düzeyine Göre Uyku Kalitesi Ve Monosit/Yüksek Yoğunluklu Lipoprotein Oranin Karşilaştirilmasi. Kırıkkale Üniv. Tıp Fakültesi Derg..

[27] Leng Y., Ahmadi-Abhari S., Wainwright N.W.J., Cappuccio F.P., Surtees P.G., Luben R., Brayne C., Khaw K.-T. (2014). Daytime napping, sleep duration and serum C reactive protein: A population-based cohort study. BMJ Open.

[28] Wang L.-X., Liu C., Shao Y.-Q., Jin H., Mao C.-J., Chen J. (2022). Peripheral blood inflammatory cytokines are associated with rapid eye movement sleep behavior disorder in Parkinson’s disease. Neurosci. Lett..

[29] Farmen K., Nissen S.K., Stokholm M.G., Iranzo A., Østergaard K., Serradell M., Otto M., Svendsen K.B., Garrido A., Vilas D. (2021). Monocyte markers correlate with immune and neuronal brain changes in REM sleep behavior disorder. Proc. Natl. Acad. Sci. USA.

[30] Purdy J.C., Shatzel J.J. (2021). The hematologic consequences of obesity. Eur. J. Haematol..

[31] Herishanu Y., Rogowski O., Polliack A., Marilus R. (2006). Leukocytosis in obese individuals: Possible link in patients with unexplained persistent neutrophilia. Eur. J. Haematol..

[32] Vera R.H., Vilahur G., Badimon L. (2013). Obesity with insulin resistance increase thrombosis in wild-type and bone marrow-transplanted Zucker Fatty rats. Thromb. Haemost..

[33] Zhang Y., Feng X., Wu X., Zhang W., Dai Y., Jiang H., Zhang X. (2022). A systematic review and meta-analysis of the relationship between erectile dysfunction and the neutrophil-to-lymphocyte and platelet-to-lymphocyte ratios. Andrologia.

[34] Akbas A., Gulpınar M.T., Sancak E.B., Gunes M., Ucar M., Altok M., Umul M. (2016). The relationship between platelet–lymphocyte ratio and severity of erectile dysfunction. Kaohsiung J. Med. Sci..

[35] Sambel M., Gulpınar M.T., Sancak E.B., Gunes M., Ucar M., Altok M., Umul M. (2018). Relationship between erectile dysfunction and the neutrophil to lymphocyte and platelet to lymphocyte ratios. Int. J. Impot. Res..

[36] Liao Z., Tang Y., Li X., Li D. (2021). The relationship between hematologic parameters and erectile dysfunction. Sex. Med..

[37] Cakir S.S., Ozcan L., Besiroglu H., Dursun M., Polat E.C., Otunctemur A., Ozbek E. (2018). Visceral adiposity index is associated with premature ejaculation inversely: A cross-sectional study. Aging Male.

[38] Clephane K., Wilson M.C., Craig A.N., Heiman J.R., Lorenz T.K. (2021). Inflammation predicts sexual arousability in healthy women. Compr. Psychoneuroendocrinol..

[39] Gastaldon C., Schoretsanitis G., Arzenton E., Raschi E., Papola D., Ostuzzi G., Moretti U., Seifritz E., Kane J.M., Trifirò G. (2022). Withdrawal Syndrome Following Discontinuation of 28 Antidepressants: Pharmacovigilance Analysis of 31,688 Reports from the WHO Spontaneous Reporting Database. Drug Saf..

[40] Cıcek E., Demırel B., Cıcek I.E., Kıraç A.S., Eren I. (2018). Increased neutrophil-lymphocyte and platelet-lymphocyte ratios in male heroin addicts: A prospective controlled study. Clin. Psychopharmacol. Neurosci..

[41] Soder H.E., Berumen A.M., Gomez K.E., Green C.E., Suchting R., Wardle M.C., Vincent J., Teixeira A.L., Schmitz J.M., Lane S.D. (2020). Elevated neutrophil to lymphocyte ratio in older adults with cocaine use disorder as a marker of chronic inflammation. Clin. Psychopharmacol. Neurosci..

[42] Sharpley C.F., Bitsika V., McMillan M.E., Jesulola E., Agnew L.L. (2019). The association between cortisol: C-reactive protein ratio and depressive fatigue is a function of CRP rather than cortisol. Neuropsychiatr. Dis. Treat..

[43] Grandner M.A., Buxton O.M., Jackson N., Sands-Lincoln M., Pandey A., Jean-Louis G. (2013). Extreme sleep durations and increased C-reactive protein: Effects of sex and ethnoracial group. Sleep.

[44] Eurelings L.S., Richard E., Eikelenboom P., van Gool W.A., van Charante E.P.M. (2015). Low-grade inflammation differentiates between symptoms of apathy and depression in community-dwelling older individuals. Int. Psychogeriatr..

[45] Hu Z.-D., Sun Y., Guo J., Huang Y.-L., Qin B.-D., Gao Q., Qin Q., Deng A.-M., Zhong R.-Q. (2014). Red blood cell distribution width and neutrophil/lymphocyte ratio are positively correlated with disease activity in primary Sjögren’s syndrome. Clin. Biochem..

